# Automated cerebral hemorrhage volume calculation and stability detection using automated software

**DOI:** 10.21203/rs.3.rs-2944493/v1

**Published:** 2023-05-23

**Authors:** Anirudh Sreekrishnan, Chitra Venkatasubramanian, Jeremy J Heit

**Affiliations:** Stanford University School of Medicine; Stanford University School of Medicine; Stanford University School of Medicine

**Keywords:** ICH, AI Detection, Imaging Detection

## Abstract

**Introduction:**

The measurement of intracerebral hemorrhage (ICH) volume is important for management, particularly in evaluating expansion on subsequent imaging. However manual volumetric analysis is time-consuming, especially in busy hospital settings. We aimed to use automated Rapid Hyperdensity software to accurately measure ICH volume across repeated imaging.

**Methods:**

We identified ICH cases, with repeat imaging conducted within 24 hours, from two randomized clinical trials where enrollment was not based on ICH volume. Scans were excluded if there was (1) severe CT artifacts, (2) prior neurosurgical procedures, (3) recent intravenous contrast, or (4) ICH < 1 ml. Manual ICH measurements were conducted by one neuroimaging expert using MIPAV software and compared to the performance of automated software.

**Results:**

127 patients were included with median baseline ICH volume manually measured at 18.18 cc (IQR: 7.31 – 35.71) compared to automated detection of 18.93 cc (IQR: 7.55, 37.88). The two modalities were highly correlated (r = 0.994, p < 0.001). On repeat imaging, the median absolute difference in ICH volume was 0.68cc (IQR: −0.60–4.87) compared to automated detection at 0.68cc (IQR: −0.45–4.63). These absolute differences were also highly correlated (r = 0.941, p < 0.001), with the ability of the automated software to detect ICH expansion with a Sensitivity of 94.12% and Specificity 97.27%.

**Conclusion:**

In our proof-of-concept study, the automated software has high reliability in its ability to quickly determine IPH volume with high sensitivity and specificity and to detect expansion on subsequent imaging.

## Introduction

Intracerebral hemorrhage (ICH) is associated with significant morbidity and mortality.^1–3^ Baseline hematoma volume^4^ and hematoma expansion (HE)^5–7^ on follow-up imaging have been identified as two key predictors of poorer clinical outcomes. Approximately 20–30% of patients will exhibit HE on follow-up imaging,^7,8^ most occurring within the first four to six hours.^9,10^ Given the acuity of these factors, prompt detection of hematoma volume as well as HE is critical for the management of this patient population.^1^

Automated software^11–13^ designed to detect ICH are becoming commonly used in clinical practice to help address potential delays in patient care. However, no automated tools have been developed specifically to assess for HE in subsequent follow-up imaging. Limitations around the identification of HE stem from the lack of standardized definitions regarding expansion or growth,^14^ as well as software infrastructure to evaluate serial imaging.

Rapid Hyperdensity (iSchemaView, Menlo Park, CA) is an FDA cleared automated software that detects the volume of hyperdense lesions in the brain. We sought to examine the feasibility of using this software for identifying HE on serial non contrast CT ICH scans. The purpose of this study was to compare manual measurement of ICH hematoma volume on serial imaging a gainst this automated software to see if there was consistency in not only the measurements of volume and growth, but also a reliability in the assessment of HE. We believe the development of an automated HE detection tool would not only aid in prognosis of patients with ICH, but could help with triage and clinical management of these patients in a busy clinical settings.

## Methods

### Image Acquisition

In this retrospective cohort study, serial NCCTs were obtained from two randomized control clinical trials (RCTs)^15^ examining the effects of therapeutic agent on the outcomes of spontaneous ICH. Inclusion criteria include all ICH scans with a baseline image as well as a follow-up image within 24 hours. Exclusion criteria for this study include: (1) severe CT artifacts, (2) prior neurosurgical procedures, (3) recent intravenous contrast, or (4) ICH < 1 ml (the specified detection limit of the software).

NCCT studies were acquired in the axial plane with slice thickness that ranged from 1 to 5 mm. Radiation doses varied by vendor and location, and these variables were not controlled for in this study, which was intended to sample variations in standard radiology practices. Images were not tilt corrected or otherwise manipulated prior to interpretation. All head CTs were processed in 5 mm thick axial slices. If CT source data were acquired with a thickness of less than 5 mm, images were merged and averaged into 5 mm thickness prior to analysis. This study was approved by the local institutional review board approval and the need for informed consent was waived.

**Figure 1:**
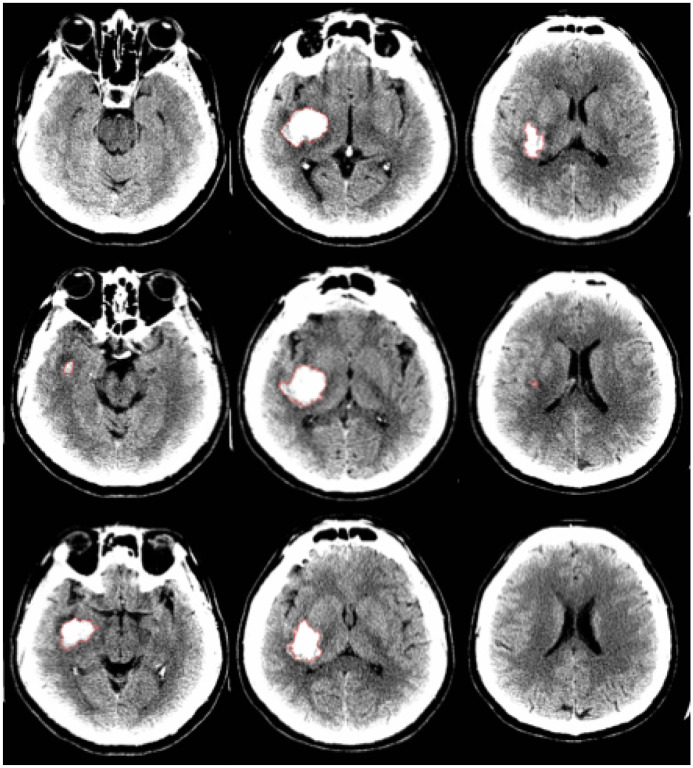
Manual measurements (in red) of hematoma used to calculate hematoma volume using MIPAV software.

Using Medical Image Processing, Analysis, and Visualization (MIPAV) software (Version 11.0.4, NIH), each ICH was manually outlined by a single imaging expert and stored as binary mask (Fig. 1). MIPAV software was used to calculate the hematoma volumes based on the binary masks created which were used as the ground truth to which the automated module was tested against.

## Automated Detection

Input into the module comprises a set of DICOM objects from a NCCT scan. Identification of parenchymal regions brighter than the normal healthy brain tissue is based on thresholding relative to the brain tissue median using a HU threshold of > 15 HU. The volume is a calculation of area of pixels identified using HU analysis multiplied by the slice thickness. Following pre-processing steps that prepare the data for hyperdense region detection, a segmentation/false positive correction algorithm is performed and then the algorithm generates summary images displaying the location of any detected hyperintensity along with the total volume of the hyperintense regions. Regions that are rejected include all slices below the brain stem, slices above the top of brain tissue/skull interface, and prominent (commonly hyperdense but not pathologic) regions of the torcular Herophili, straight sinus, inferior sagittal sinus, transverse sinus, superior sagittal sinus and the falx cerebri. The processing time to detect ICH volume is typically about 1 minute.

### Statistical Analysis

Descriptive statistics were used to evaluate the hematoma volume of the baseline and follow-up imaging for both the manual measurements and the automated software. Hematoma growth was evaluated by calculating the absolute difference, subtracting the follow-up hematoma volume from the baseline volume, as well as the percent difference, dividing the absolute difference by the baseline volume. Comparisons between manual measurement and the software was conducted by calculating a Pearson correlation.

HE was defined similarly to previous studies,^14^ by looking at both a 6cc increase as well as a 33% increase in hematoma volume from baseline imaging. The decision was made to use both criteria in conjunction, rather than either/or, to increase the specificity of identifying HE in our cohort. Using these criteria, all scans were classified into positive/negative HE based on the manual measurements as well as the software. The accuracy, sensitivity, specificity, positive predictive value, negative predictive value, and positive and negative likelihood ratios of detecting HE by the software were calculated for the purposes of this analysis. All calculations were conducted using SPSS v.28 software (SPSS Inc., Chicago, Illinois, USA).

## Results

**Figure 2:**
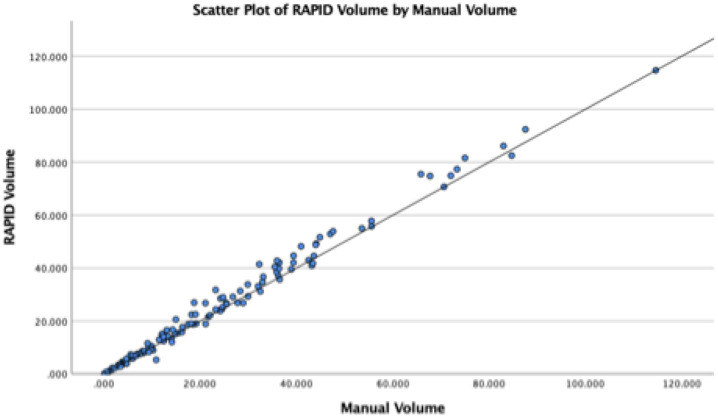
Correlation of manual measurements of hematoma volume (cc) compared to automated software calculation for baseline imaging. Pearson correlation: 0.994, p < 0.001.

A total of 127 patients with both baseline and follow-up imaging were included in this analysis; baseline characteristics of this patient cohort are included in Table 1. A full breakdown of hematoma volume measurements and evaluation of growth is included in Table 2. Median hematoma volume on baseline imaging was measured at 18.18cc (IQR: 7.31–35.71) compared to software evaluation of 18.93cc (IQR: 7.55, 37.88). Baseline hematoma volume was highly correlated (Fig. 2) between automated and manual measurements (r = 0.994, p < 0.001).

Follow-up imaging showed a slightly larger hematoma volume, with the median increase in hematoma volume measured at 0.68cc (IQR: −0.60–4.87) and 0.68cc (IQR: −0.45–4.63) across manual and software measurements respectively, which was also highly correlated (r = 0.941, p < 0.001). The median percentage increase in hematoma growth was also similarly correlated at 6.04% (IQR: −5.49–26.19) and 5.28% (IQR: −2.42–25.30) across manual and automated measurements respectively (r = 0.976, p < 0.001).

The ability to identify HE, defined as both an increase in hematoma volume by 6cc as well as a 33% increase, was compared between radiology detection and the software and displayed in Table 3. Compared to radiology detection, the automated software was able to identify HE with a sensitivity of 94.12% (CI: 71.31–99.85) and specificity of 97.27% (CI: 92.24–99.43). This resulted in a positive likelihood ratio of 34.51 (CI: 11.23–106.02) and a negative likelihood ratio of 0.06 (CI: 0.01–0.41).

## Discussion

In this proof-of-concept study, we have been able to show that it is not only feasible to use automated tools to have strong internal consistency with hematoma volume calculation, but also reliably assess for HE on serial imaging. While identification of ICH has been shown to be achievable with automated software,^11–13^ this is the first study to add a new dimension and FDA-cleared volume calculation that can be used for assessment of HE across serial imaging studies. Specifically, in regards to HE, we show a fairly high sensitivity and specificity in this pilot data using already established definitions of HE in the literature.

Though focused on addressing the feasibility of this approach, this analysis was strengthened by using real-world cases as well as having all manual measurements conducted by one reviewer. This methodology helped strengthen the generalizability of our results as well as eliminated any potential inter-rater reliability within hematoma measurements. Similarly, the inclusion of RCT scans was conducted to help attain an appropriate sample size for this analysis. There is a potential confounder with the impact of the therapeutic intervention on HE, however our analysis was not interested in the absolute rates of HE but rather the ability to identify HE on serial imaging.

Another aspect of our approach was the identification of patients that exhibited a ‘negative expansion,’ or patients where the difference between baseline hematoma volume and the subsequent volume resulted in a negative value. These cases routinely occurred with ICHs in the brainstem where slice thickness and image acquisition can result in slight differences in which slices show most of the hematoma. However, manual review of these cases failed to result in the identification of a patient with true HE that was masked due to issues of imaging acquisition.

The generalizability of these results stems from the definitions of HE used for this analysis. There is no consistency in the literature regarding how HE is defined,^14^ with some studies using an absolute volume increase threshold (i.e. 6cc increase) or percentage increase threshold (i.e. 33% increase) or a combination of both rules. Additionally, there is no consensus on a threshold that qualifies as a clinically meaningful increase, only that the presence of HE suggests a poorer prognosis.^5–7^ Given the inconsistency in the field, we specifically choose the most widely used thresholds and used both rules in conjunction to help increase the specificity of identification.

When applying our method of HE to clinical management, our approach decreases false positive identification and would potentially highlight the highest risk patients showing expansion. With new automated tools being developed to predict HE,^16^ we will need to have concurrent tools to reliably identify HE in the field. Automated software can potentially serve to appropriately highlight patients with expansion in order to tailor therapeutic interventions or triage patients for closer monitoring. Especially in busy medical centers, automated software can aid in the rapid identification of these at-risk patients as well as provide prognostic insight that might not be achievable without specialized subspecialty care.

## Figures and Tables

**Table 1:** Baseline characteristics of the total cohort.

	Total Cohort n = 127
Age, mean years (IQR)	61 (50–75)
NIHSS, median (IQR)	12 (4–17)
GCS, median (IQR)	14 (10–15)
Female Gender, n (%)	46 (36.2)
*Race*, n (%)	
White	89 (70.1)
Black	13 (10.2)
Asian	23 (18.1)
Missing	2 (1.6)
Hispanic, n (%)	28 (22.1)
*Past Medical History*, n (%)	
Hx of HTN	92 (72.4)
Hx of Diabetes	27 (21.3)
Hx of Stroke/TIA	20 (15.8)
On Anti-HTN medications	71 (55.9)
Medication Compliance	40 (31.5)

**Table 2:** Hematoma volume and calculation of hematoma growth for manual measurements compared to automated measurement.

Volume Metric	Manual Measurement	Automated Measurement
*Baseline Scans*		
Mean Volume, cc	23.90	25.42
Median Volume, cc	18.18	18.93
IQR, cc	7.31, 35.71	7.55, 37.88
*Follow-up Scans*		
Mean Volume, cc	27.43	29.24
Median Volume, cc	22.65	23.46
IQR, cc	9.24, 37.23	9.53, 38.86
*Absolute Difference*		
Mean Volume, cc	3.53	3.82
Median Volume, cc	0.68	0.68
IQR, cc	−0.60, 4.87	−0.45, 4.63
*Percentage Difference*		
Mean Percentage, %	160.26	21.97
Median Percentage, %	6.04	5.28
IQR, %	−5.49, 26.19	−2.42, 25.30

**Table 3:** Comparison of ICH hematoma expansion detection by radiologist vs Automated detection.

		Radiologist Detection	
		ICH Growth	ICH Non-growth
**Automated Detection**	ICH Growth	16	3
	ICH Non-growth	1	107
